# A systematic review of the diagnostic accuracy of automated tests for cognitive impairment

**DOI:** 10.1002/gps.4852

**Published:** 2018-01-22

**Authors:** Rabeea'h W. Aslam, Vickie Bates, Yenal Dundar, Juliet Hounsome, Marty Richardson, Ashma Krishan, Rumona Dickson, Angela Boland, Joanne Fisher, Louise Robinson, Sudip Sikdar

**Affiliations:** ^1^ Health Services University of Liverpool Liverpool UK; ^2^ Mersey Care NHS Foundation Trust Liverpool UK; ^3^ Institute of Health and Society Newcastle University Newcastle upon Tyne UK

**Keywords:** ageing, Alzheimer disease, automated tests, computerised tests, dementia, diagnosis, MCI, monitoring

## Abstract

**Objective:**

The aim of this review is to determine whether automated computerised tests accurately identify patients with progressive cognitive impairment and, if so, to investigate their role in monitoring disease progression and/or response to treatment.

**Methods:**

Six electronic databases (Medline, Embase, Cochrane, Institute for Scientific Information, PsycINFO, and ProQuest) were searched from January 2005 to August 2015 to identify papers for inclusion. Studies assessing the diagnostic accuracy of automated computerised tests for mild cognitive impairment (MCI) and early dementia against a reference standard were included. Where possible, sensitivity, specificity, positive predictive value, negative predictive value, and likelihood ratios were calculated. The Quality Assessment of Diagnostic Accuracy Studies tool was used to assess risk of bias.

**Results:**

Sixteen studies assessing 11 diagnostic tools for MCI and early dementia were included. No studies were eligible for inclusion in the review of tools for monitoring progressive disease and response to treatment. The overall quality of the studies was good. However, the wide range of tests assessed and the non‐standardised reporting of diagnostic accuracy outcomes meant that statistical analysis was not possible.

**Conclusion:**

Some tests have shown promising results for identifying MCI and early dementia. However, concerns over small sample sizes, lack of replicability of studies, and lack of evidence available make it difficult to make recommendations on the clinical use of the computerised tests for diagnosing, monitoring progression, and treatment response for MCI and early dementia. Research is required to establish stable cut‐off points for automated computerised tests used to diagnose patients with MCI or early dementia.

## 1 INTRODUCTION

Cognitive impairment in dementia is a growing public health concern.^1^ It is a distinctive characteristic of all dementias, and its timely assessment is a crucial and essential element in the diagnosis of dementia.^2^ This is because some causes of dementia are treatable and are fully or partially reversible, including dementias caused by vitamin B_12_ deficiency,^3^ side effects of medications,^4^ metabolic abnormality, and certain brain tumours.^5^ There is evidence from the United States that early recognition and treatment of dementia may delay the subsequent need for nursing home care and may reduce the risk of misdiagnosis and inappropriate management and reduce responsibilities for carers.^6^

Obtaining accurate incidence and prevalence figures for MCI is difficult since people with cognitive impairment may go undiagnosed. These estimates also vary significantly depending on the definitions used in different studies. For example, a large population‐based study of older‐aged individuals in the United Kingdom^7^ reported prevalence estimates of individuals not classified from current MCI definitions were variable (range, 2.5‐41.0%). In addition, the rates of progression from MCI to dementia varied from 3.7% to 30.0%.^7^

Evidence from neuropathological and neuroimaging studies suggests that biological changes associated with dementia occur long before the onset of symptoms.^8^ This has given rise to the concept of mild cognitive impairment (MCI), which is the state between the cognitive changes of normal ageing and early dementia.^9^, ^10^, ^11^ Mild cognitive impairment refers to the clinical condition used to describe people whose cognitive function is below that of the normal population for their educational level and age but who do not have any loss of functional abilities or skills.^11^, ^12^, ^13^, ^14^ It is a heterogeneous state, with possible trajectories including Alzheimer disease (AD), Lewy body dementias, and even reversion to normal cognitive functioning.^15^

The difference between MCI and early dementia is based on the level of cognitive decline and pattern of change in mood and behaviour. Individuals diagnosed with early dementia present with multiple cognitive deficits, and their memory loss is sufficient to impact everyday social and occupational functioning. Among the 4 most common medical conditions causing dementia are AD, vascular conditions, frontotemporal atrophy, and Lewy body disease. Irrespective of the primary reason, the cognitive prognosis for people with most types of dementia is usually poor.^16^, ^17^

There are a number of pen‐and‐paper–based tools as suitable tests for screening people for cognitive impairment, for example, the General Practitioner Assessment of Cognition, 6‐item Cognitive Impairment Test, and Mini‐cog assessment instrument.^18^, ^19^ There are different pen‐and‐paper tests used to aid diagnosis by specialists for MCI and early dementia, for example, the Dementia Toolkit for Effective Communication,^20^ Montreal Cognitive Assessment,^21^ and Saint Louis University Mental Status.^22^ However, these specialist tests can be expensive and time‐consuming.^23^ More recently, several automated tests have been developed,^24^, ^25^ which may be uniquely suited to early detection of changes in cognition, by, for example, covering a wider range of ability to precisely record accuracy and speed of response with a level of sensitivity not possible in standard administrations.^23^

The rationale for this review is to determine whether automated computerised tests for cognitive impairment have the potential to contribute to early diagnosis and simplify the current method of monitoring progression and treatment response compared with standard clinical practice.

**Key points**

- Timely diagnosis of mild cognitive impairment (MCI) and early dementia is important for good prognosis and effective management.
- A number of automated tests for diagnosing and monitoring progression of cognitive impairment have been developed, which need to be used in conjunction with clinical assessment.
- The overall quality and quantity of the available evidence are insufficient to make recommendations on the clinical use of these automated computerised tests.
- Further research is required to examine the cut‐off points for different populations in automated tests for diagnosing and monitoring progression and treatment response of MCI and early dementia.

## 2 METHODS

A systematic review was performed to describe the diagnostic accuracy of automated tests to detect MCI and early dementia as well as investigate their role in monitoring disease progression and response to treatment. The methodology and reporting of this review followed the guidance set out by the Cochrane Handbook for Diagnostic Test Accuracy Reviews.^26^ See Appendix S1 found in the Supporting Information for an abbreviation list.

### 2.1 Criteria for considering studies for this review

Any study assessing the diagnostic accuracy of automated computerised tests to diagnose or monitor MCI or early dementia against a reference standard was considered for inclusion. Case studies and qualitative studies were excluded. Studies or diagnostic tools published in a non‐English language were also excluded.

#### 2.1.1 Participants

Participants were people with MCI or early dementia diagnosed by any recognised diagnostic standard.

#### 2.1.2 Index tests

The index tests considered for inclusion were automated computerised tests of cognitive impairment, which can either be self‐administered or interviewer administered.

#### 2.1.3 Reference standard

The reference standard for this review is the clinical diagnosis of MCI and early dementia using a diagnostic criteria, for example, the *International Classification of Diseases* 2 edition 10 and the *Diagnostic and Statistical Manual of Mental Disorders* editions 4 and 5 (DSM‐IV and DSM‐V, respectively).^27^ It is recognised that clinical diagnosis itself has a degree of variability, but this is not unique to dementia studies and does not invalidate the basic diagnostic test accuracy approach.

### 2.2 Search methods for identification of studies

The following electronic databases were searched from January 2005 to August 2015 to identify studies for inclusion: Medline, Embase, Cochrane database, Institute for Scientific Information, PsycINFO, and ProQuest for dissertations and theses (see Appendix S2 found in the Supporting Information for search strategy in Medline). Through citation tracking, one study from 2001 was included since it reported on a computerised tests currently in use in clinical practise. The number of references retrieved from different databases is provided in Appendix S3 found in the Supporting Information, and were managed in Endnote X7.

### 2.3 Selection of studies

Two reviewers independently screened all relevant titles and abstracts and full‐text articles for inclusion. Any disagreements were resolved by discussion with a third reviewer.

### 2.4 Data extraction and management

Data extraction forms were developed and piloted in an Excel spreadsheet by using 2 of the included studies. Data on study design, population characteristics, and outcomes were extracted by one reviewer and independently checked for accuracy by a second reviewer, with disagreements resolved through discussion with a third reviewer when necessary. The extracted data included information on the reference standard, index test, cut‐off points, and the measures of diagnostic test accuracy including sensitivity, specificity, receiver operating characteristic curve, and the area under the curve (AUC) for discriminating amongst MCI, early dementia, and cognitively healthy individuals.

### 2.5 Assessment of methodological quality

The methodological quality of the included studies was assessed by one reviewer and independently checked for accuracy by a second reviewer using the Quality Assessment of Diagnostic Accuracy Studies tool,^28^ which is recommended by the Cochrane Diagnostic Test Accuracy Reviews Guidelines.^29^ This tool is designed to evaluate the risk of bias and applicability of primary diagnostic accuracy studies using signalling questions in 4 domains: patient selection, index test, reference standard, and flow and timing.

### 2.6 Statistical analysis and data synthesis

An Excel spreadsheet was used to construct 2 × 2 tables of index test performance. The measures of index test performance were recorded by the number of true‐positive, true‐negative, false‐positive, and false‐negative, sensitivity, and specificity values of MCI and early dementia. The sensitivity and specificity values with 95% confidence intervals, positive and negative predictive values (PPV and NPV, respectively), and positive and negative likelihood ratios (LR+ and LR−, respectively) were calculated when not reported in the studies. Out of authors of all the included studies approached with a request for specific sensitivity and specificity data, only 2 provided these data.

It was not possible to perform a meta‐analysis because of noncomparable data; the study designs varied, the cut‐off points for the primary outcome measure were heterogeneous, and the summary statistics were often inconsistently reported. A narrative synthesis of the results of the included studies was conducted.

### 2.7 Patient and public involvement

An advisory group comprising clinicians and service users guided the team during the review. A call for participation was sent through frontline groups, for example, Alzheimer's Society and Dementia UK, to identify people interested in giving feedback on the results of the review and on the final report. The review team took guidance from these agencies and INVOLVE^30^ for planning and facilitating the meetings.

## 3 RESULTS

### 3.1 Results of the search

**Figure 1:**
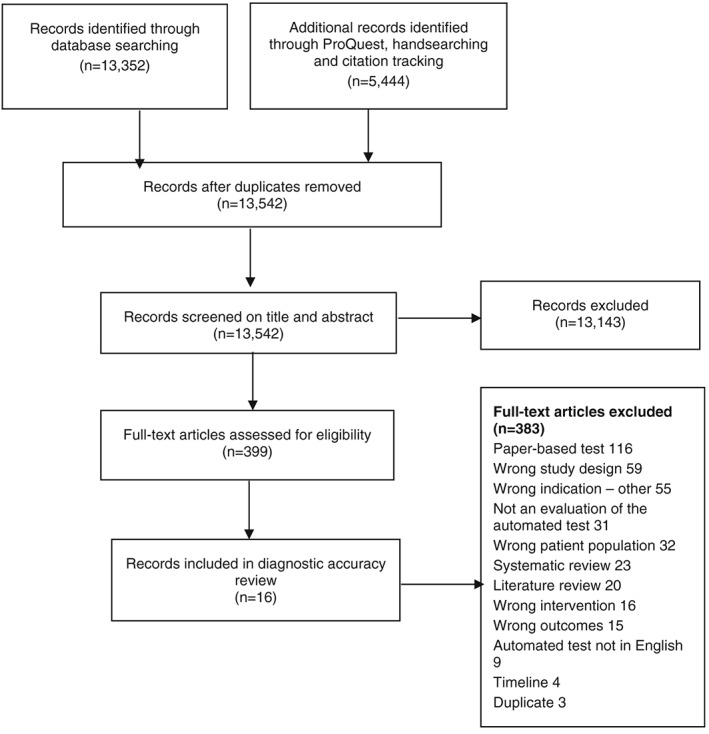
Preferred Reporting Items for Systematic Reviews and Meta‐Analyses flow diagram

The electronic search was conducted in August 2015, and 18 796 records were retrieved, of which 399 articles were shortlisted for full‐text assessment (Figure 1). The comprehensive search strategy was necessary because indexing of diagnostic accuracy studies is poor. In total, 16 studies met the inclusion criteria for detecting MCI and early dementia. No studies met the review inclusion criteria for monitoring progression or treatment response in MCI or early dementia, and therefore, there is no further mention of monitoring disease progression in the results section.

In addition to the 16 included studies, 4 trials were identified during hand searching (Appendix S4 found in the Supporting Information). The authors of these studies were approached by email and telephone for results, but no responses were received. The summary of the included 16 studies is presented in Table 1; there were 7 cohort studies, 7 case‐control studies, and 2 cross‐sectional studies.^40^^,^ 43 Seven of the 16 included studies evaluated the use of automated computerised tests to detect MCI alone, 2 studies reported results for early dementia, 6 studies reported results for combined MCI/early dementia, and 1 study reported on cognitive impairment with a co‐morbidity, eg, human immunodeficiency virus (HIV)–associated neurocognitive disorders (HANDs).^43^ Two different reference standards were used for MCI in these studies, 9 studies used the Petersen criteria, and 4 studies used clinical diagnosis with a battery of neurocognitive tests. The reference standard for early dementia varied across different studies, 2 studies used National Institute of Neurological and Communicative Disorders and Stroke and the Alzheimer's Disease and Related Disorders Association Alzheimer's Criteria,^42^, ^46^ 2 studies used DSM‐IV,^33^, ^34^ 1 study used the DSM‐V criteria,^39^ 2 studies used clinical diagnosis with neurocognitive tests,^36^, ^46^ and 1 study used the Clinical Dementia Rating score.^41^

**Table 1:** Study and participant characteristics

Study	Condition	Country, Setting	N	Mean age, years (SD, range)	Gender (Male %)	Mean Education, y (SD, Range)	Index Test Name	Reference Test
Ahmed et al^31^	MCI	United Kingdom	35 (control: 20, MCI: 15)	Control: 77.4 (4)	Control: 55.0	Control: 14.7 (2.9)	CANS‐MCI	Clinical diagnosis using the Petersen criteria
		Primary care (Oxford OPTIMA study)^a^		MCI: 80.9 (7.2)	MCI: 33.3	MCI: 13.1 (3)		
De Jager et al^32^	MCI	United Kingdom	119 (control: 98, MCI: 21)	Control: 77.18 (5.9)	NR	Unclear	CogState	Clinical diagnosis using battery of neurocognitive tests
		Community		MCI: 81.95 (5.4)				
Doniger et al^33^	MCI	United States	161 (control: 71, MCI: 58, mild AD: 32)	Entire group: 76.0 (8.2)	Entire group: 37.5	Entire group: 13.3 (3.6)	Mindstreams (abridged)	Clinical diagnosis using the Petersen criteria for MCI and DSM‐IV for dementia
		Tertiary care						
	MCI/mild dementia	Memory clinic						
Dwolatzky et al^34^	MCI	Canada/Israel	98 (control: 39, MCI: 30, mild AD: 29)	Control: 73.41 (8.00)	Control: 33.3	Control: 14.95 (3.5)	Mindstreams	Clinical diagnosis using the Petersen criteria for MCI and DSM‐IV for mild AD
	Mild AD	2 tertiary care memory clinics		MCI: 77.15 (6.43)	MCI: 56.7	MCI: 13.07 (2.86)		
				Mild AD: 80.55 (4.91)	Mild AD: 44.8	Mild AD: 11.31 (2.85)		
Juncos‐Rabadan et al^35^	aMCI	Spain	162 (control: 85, mda‐MCI: 29, sda‐MCI: 48)	Control: 62.25 (8.26, 50‐82)	All participants: 36.4	Control: 10.83 (5, 2‐21)	CANTAB‐R (PRM, DMS, and PAL)	Clinical diagnosis using neurocognitive tests and the Albert criteria and Peterson criteria for aMCI
		Primary care		mda‐MCI: 71.68 (7.74, 54‐87)		mda‐MCI: 10.06 (3.99, 3‐20)		
				sda‐MCI: 68.02 (9.04, 50‐84)		sda‐MCI: 9.83 (3.96, 2‐20)		
Junkkila et al^36^	aMCI/mild/probable dementia	Finland	58 (control: 22, aMCI: 17, AD: 19)	Control: 70 (4.48, 65‐80)	Control: 36.36	Control: 10 (3.25)	CANTAB‐PAL	Clinical diagnosis using the Petersen criteria and neurocognitive tests
	Mild/probable dementia	Hospital		aMCI: 73 (6.3, 61‐83)	aMCI: 64.7	aMCI: 8 (3)		
				AD: 73 (6.76, 61‐83)	AD: 26.35	AD: 8 (2.88)		
Kingsbury et al^37^	MCI	Australia	140 (control: 95, MCI: 30, depressed: 15)	Control: 68.85 (7.96, 53‐89)	Control: 37	Controls: 4.93 (1.71)	CogniScreen	Clinical diagnosis using the Petersen criteria
		Community		MCI: 77.62 (7.45, 51‐87)	MCI: 43	MCI: 3.07 (1.71)		
		Memory clinic				Unclear what is measured		
Kluger et al^38^	MCI	United States	101 (control: 39, MCI: 19, probable AD: 17, no diagnosis: 25)	Control: 64 (11)	NR	NR	Computerised test (no name)	Diagnosed by a consensus of at least 2 clinicians
	Early dementia	Memory clinic		MCI: 72 (10)				
				Probable AD: 78 (9)				
Lichtenberg et al^39^	MCI/early dementia	United States	102 (control: 55, MCI: 11, mild dementia: 36)	All participants: 79.3 (6.6)	All participants: 46.1	All participants: 13.5 (2.9)	CST	Clinical diagnosis using the Petersen criteria; clinical diagnosis of dementia using DSM‐V
		Specialised geriatric clinic						
Maruff et al^40^	MCI	Australia	766 (control: 659, aMCI: 107)	Control: 69.5(6.6)	Control: 42.2	Control: 12^a^ (9‐15)	CBB	Clinical diagnosis using the Peterson criteria
		Primary care		MCI: 75.7 (7.5)	MCI: 49.5	MCI: 12^a^ (9‐15)		
Mundt et al^41^	Dementia	United States	116 (control: 74, mild dementia: 42)	All participants: 76.7 (7.0, 56‐93)	All participants: 36.7	All participants: 13.3 (3, 6‐22)	Computer‐automated telephone screening	Clinical diagnosis using CDR score
		Specialised geriatric clinic						
O'Connell et al^42^	Probable AD	Ireland	50 (control: 16, probable AD: 34)	Control: 72.6 (7.7)	Control: 12.5	NR	CANTAB‐PAL	Clinical diagnosis using the NINCDS‐ADRDA criteria
		Memory clinic		Probable AD: 73 (5.9)	Probable AD: 32.4			
Rosenthal et al^43^	HAND	United States	55 (HIV+ controls:16, HAD: 39)	HIV+ controls: 45.4 (6)	HIV+ controls: 75.0	HIV+ controls: 12.3 (1.8)	CAMCI modified	HAND category using the Frascati criteria
		General clinical research clinic		HAD: 48.3 (6.3)	HAD: 71.8	HAD: 12.6 (2.1)		
Saxton et al^44^	MCI	United States	524 (control: 296, MCI: 228)	Control: 71.84 (5.95)	MCI: 37.7	Control 13.74 (2.69)	CAMCI	Clinical diagnosis by consensus using battery of neurocognitive tests and functional and medical information
		Primary care and community		MCI: 75.18 (6.76)	Control: 32.8	MCI: 13.10 (2.61)		
Tierney et al^45^	MCI	Canada	263	Completed without assistance: 78.7 (6.9)	All participants: 41.4	Completed without assistance: 15.2 (3.2)	CAMCI	Clinical diagnosis using battery of neurocognitive tests
		Tertiary care	NR	Completed with assistance: 81.8 (6.5)		Completed with assistance: 13.9 (4.0)		
Vacante et al^46^	MCI	United Kingdom	78 (control: 40, MCI: 20, early AD: 18)	*Traditional version*	*Traditional version*	*Traditional version*	TPT	Clinical diagnosis using the Petersen criteria
				Control: 74.7 (7.78)	Control: 50	Control: 15.85 (3.36)		
				MCI: 78.3 (8.4)	MCI: 60	MCI: 15.9 (3.32)		
				Early AD: 73.67 (6.28)	Early AD: 66.7	Early AD: 15 (3.04)		
	Early dementia	Primary care (Oxford OPTIMA study)^a^		*Novel version*	*Novel version*	*Novel version*		
				Control: 73.67 (7.14)	Control: 45	Control: 16.35 (3.18)		
				MCI: 79.7 (6.07)	MCI: 60	MCI: 15 (2.66)		
				Early AD: 77.22 (4.94)	Early AD: 77.8	Early AD: 16.11 (2.97)		

Abbreviations: AD, Alzheimer disease; aMCI, amnestic mild cognitive impairment; CAMCI, Computer Assessment of Mild Cognitive Impairment; CANS‐MCI, Computer‐Administered Neuropsychological Screen for Mild Cognitive Impairment; CANTAB, Cambridge Neuropsychological Test Automated Battery; CANTAB‐PAL, Cambridge Neuropsychological Test Automated Battery Paired Associated Learning; CBB, CogState Brief Battery; CDR, Clinical Dementia Rating Scale; CST, Computerised Self‐Test; DMS, Delayed Matching to Sample; DSM‐IV, *Diagnostic and Statistical Manual of Mental Disorders* edition 4; HAD, HIV‐associated dementia; HAND, HIV‐associated neurocognitive disorder; HIV+, human immunodeficiency virus; NR, not reported; MCI, mild cognitive impairment; mda‐MCI, multiple‐domain amnestic mild cognitive impairment; NINCDS‐ADRDA, National Institute of Neurological and Communicative Disorders and Stroke and the Alzheimer's Disease and Related Disorders Association; OPTIMA, Oxford Project to Investigate Memory and Ageing; PAL, Paired Associated Learning; PRM, Pattern Recognition Memory; sda‐MCI, single‐domain amnestic mild cognitive impairment; TPT, The Placing Test.

a It is unclear as to whether these cohorts were independent to each other.

Median.

#### 3.1.1 Findings

The diagnostic accuracy of 11 automated computerised tests for the detection of MCI and/or early dementia without co‐morbidities was evaluated in 15 studies and 1 study with co‐morbidity.^43^ The details of the index tests are summarised in Table 2. Pooling of data from these 16 studies was considered inappropriate since there were few studies evaluating the same index test in the same population, and it was only possible to extract 2 × 2 data from 5 of the 16 studies.

**Table 2:** Index test details

Study	Test Name	Cognitive Domains Tested	Details of Test Platform Used	Time (min)	Method of Administration
Ahmed et al^31^	CANS‐MCI	Memory	Desktop computer, a touch screen system with both oral (loud speakers) and on screen instructions	30	Self‐administered
		Language			
		Visuospatial			Researcher in room
		Executive function			
De Jager et al^32^	CogState	Memory	Internet	Approximately 20	Self‐administered
		Executive function			
		Attention			Practice session with a psychologist
		Processing speed			
Doniger et al^32^	Mindstreams (abridged)	Memory	Computer and mouse	30	Self‐administered
		Executive function			
		Visuospatial			Practice session
		Motor skills			
Dwolatzky et al^34^	Mindstreams	Memory	Designed for use with older people. Mouse with the number pad on the keyboard (similar to the telephone keypad)	45	Self‐administered
		Executive function			Practice session with feedback prior to testing
		Visuospatial			
		Verbal			Research assistant
		Attention			
		Information processing			
		Motor skills			
Juncos‐Rabadan et al^35^	CANTAB‐R (PRM, DMS, and PAL)	Memory	Touch screen computer	NR	Self‐administered
					Researcher present
Junkkila et al^36^	CANTAB‐PAL	Memory	Touch screen computer	NR	Self‐administered
Kingsbury et al^37^	CogniScreen	Memory	Laptop, headset with microphone	20‐40	Self‐administered
					Experimenter in room
Kluger et al^38^	Computerised test (no name)	Memory	Laptop	12‐15	Self‐administered
		Praxis			
		Naming			Screening test for computer competency
		Executive function			
Lichtenberg et al^39^	CST	Learning	Internet based, interface with both written and oral instructions	15	Self‐administered
		Memory			Keyboard proficiency test
		Executive function			Administered by graduate psychology students
Maruff et al^40^	CBB	Memory	Desktop computer, yes/no button attached through USB port	10	Self‐administered
					Verbal instructions by supervisor
					Practice session
Mundt et al^41^	Computer‐automated telephone screening	Memory	Standard touch tone telephones	11‐15	Self‐administered
		Spatial (auditory)			
		Executive function orientation			Researcher provided assistance in dialling the number
		Language			
O'Connell et al^42^	CANTAB‐PAL	Memory	Touch screen computer	10	NR
Rosenthal et al^43^	CAMCI modified	Memory	Tablet with stylus	25	Self‐administered
		Attention			
		Executive function processing speed			
Saxton et al^44^	CAMCI	Memory	Desktop computer	Approximately 20	Self‐administered
		Attention			
		Executive function processing speed			
Tierney et al^45^	CAMCI	Memory	Tablet computer	30	Self‐administered, some required researcher assistance
		Attention			
		Executive function processing speed			
Vacante et al^46^	TPT	Memory	Computer	20	Self‐administered
					Including practice pages

Abbreviations: CAMCI, Computer Assessment of Mild Cognitive Impairment; CANS‐MCI, Computer‐Administered Neuropsychological Screen for Mild Cognitive Impairment; CANTAB, Cambridge Neuropsychological Test Automated Battery; CANTAB‐PAL, Cambridge Neuropsychological Test Automated Battery Paired Associated Learning; CBB, CogState Brief Battery; CST, Computerised Self‐Test; DMS, Delayed Matching to Sample; NR, not reported; PAL, Paired Associated Learning; PRM, Pattern Recognition Memory; TPT, The Placing Test.

### 3.2 Studies reporting on diagnostic accuracy outcomes with a 2 × 2 table

There were 5 studies that reported diagnostic accuracy outcomes in a 2 × 2 table as described in Table 3. Two studies reported the diagnostic accuracy outcomes for MCI, 3 studies reported outcomes for early dementia, and 1 study reported combined outcomes for both MCI and early dementia.

**Table 3:** Diagnostic accuracy outcomes with 2 × 2 table

Study	Index Test	Cut‐off	Sensitivity, %	Specificity, %	AUC	TP	FN	TN	FP	PPV, %	NPV, %	LR +	LR−
MCI													
Juncos‐Rabadan et al^35^	CANTAB												
	Overall^a^		79.7	76.3	NR	55	14	71	22	71.4	83.3	3.4	0.3
	PRM	1.5 SD below controls	45.5^b^	92.9^b^	0.704^b^	35	42	79	6	85.4^b^	65.3^b^	6.44^b^	0.59^b^
	DMS	1.5 SD below controls	23.4^b^	97.6^b^	0.623^b^	18	59	83	2	90.0^b^	58.5^b^	9.94^b^	0.78^b^
	PAL	1.5 SD below controls	58.4^b^	89.4^b^	0.747^b^	45	32	76	9	83.3^b^	70.4^b^	5.52^b^	0.46^b^
Saxton et al^44^	CAMCI	Final tree model	86	94	0.91^b^	201	27	277	19	91.4^b^	91.1^b^	13.7^b^	0.127^b^
Early dementia													
Junkkila et al^36^	CANTAB‐PAL	NR	81.8^b^	97.2^b^	0.914^b^	18	4	35	1	94.7^b^	89.7^b^	5.35^b^	0.0.3^b^
Mundt et al^41^	Computer‐automated telephone system	A derived scoring algorithm	79.17^b^	83.8^b^	0.819^b^	38	10	62	12	76.0^b^	86.1^b^	4.88^b^	0.249^b^
O'Connell et al^42^	CANTAB‐PAL	32 errors	67.6	100	0.780	23	11	16	0	100	59.3^b^		0.324
MCI/early dementia													
Junkkila et al^36^	CANTAB‐PAL	NR	96.9^b^	80.8^b^	0.897^b^	31	1	21	5	86.1^b^	95.5^b^	5.04^b^	0.04^b^

Abbreviations: AUC, area under curve; CAMCI, Computer Assessment of Mild Cognitive Impairment; CANTAB, Cambridge Neuropsychological Test Automated Battery; CANTAB‐PAL, Cambridge Neuropsychological Test Automated Battery Paired Associated Learning; DMS, Delayed Matching to Sample; FN, false negative; FP, false positive; LR−, negative likelihood ratio; LR+, positive likelihood ratio; MCI, mild cognitive impairment; NPV, negative predictive value; NR, not reported; PAL, Paired Associated Learning; PPV, positive predictive value; PRM, Pattern Recognition Memory; TN, true negative; TP, true positive.

a The study details were provided by the primary author.

b Calculated by the research team.

#### 3.2.1 Mild cognitive impairment

Juncos‐Rabadan et al^35^ evaluated 3 different visual episodic memory tests included in the Cambridge Neuropsychological Test Automated Battery (CANTAB); these memory tests were Pattern Recognition Memory, Delayed Matching to Sample, and Paired Associated Learning. The overall sensitivity and specificity for the 3 visual episodic memory tests were moderate at 79.7% and 76.3%, respectively. The overall AUC for the different visual episodic tests was not reported, but ranged from 0.623 (Delayed Matching to Sample) to 0.747 (Paired Associated Learning), showing poor ability to discriminate between the MCI group and the non‐MCI group. This test had a high overall PPV of 71.4%; this means 71.4% of the people who tested positive for MCI with the index test actually had MCI according to the reference standard. Similarly, the overall NPV for this test was 83.3%, meaning that 83.3% of people who tested negative for MCI on the index test did not have MCI. This test had a low overall LR+ of 3.4, which shows a low likelihood of the test to establish the presence of disease. It also had a low overall LR− of 0.3, which shows a low likelihood of the test to establish the absence of disease.

The study by Saxton et al^44^ evaluated the Computer Assessment of Memory and Cognitive Impairment (CAMCI) and reported good sensitivity (86%) and exceptional specificity (94%). The reported AUC (0.91) was also very high.

#### 3.2.2 Early dementia

The CANTAB Paired Associated Learning (CANTAB‐PAL) was evaluated in 2 of the studies. Junkikla et al^36^ reported high sensitivity (81.8%) and specificity (97.2%) and an AUC of exceptional discrimination (0.914) for early dementia.

The study by O'Connell et al^42^ reported poor sensitivity (67.6%) and high specificity (100%) and an AUC of moderate discrimination (0.780) between the early‐dementia group and non–early‐dementia group.

Mundt et al^41^ assessed the Computer Automated Telephone System and reported moderate sensitivity (79.17%) and high specificity (83.8%) for this test.

#### 3.2.3 MCI/early dementia

One study evaluated CANTAB‐PAL. The authors reported high sensitivity (96.9%) and high specificity (80.8%) with an AUC of good discrimination (0.897) between the MCI/early‐dementia group and non‐MCI/early‐dementia group.

### 3.3 Studies reporting on diagnostic accuracy outcomes without a 2 × 2 table

The authors of 11 studies reported diagnostic accuracy outcomes for 9 different index tests without using 2 × 2 data as tabulated in Table 4. Instead, they calculated optimal sensitivity and specificity values using receiver operating characteristic curve analysis.

**Table 4:** Diagnostic accuracy outcomes without 2 × 2 table

Study	Index Test	Cut‐off	Sensitivity, %	Specificity, %	AUC (95% CI)	PPV, %	NPV, %	LR+	LR−
MCI									
Ahmed et al^31^	CANS‐MCI	0.5	89.0	73.0	0.867 (0.743‐0.990)	60	84	NR	NR
De Jager et al^32^	CogState								
	Accuracy	82.6	78.0	90.0	0.86 (NR)	NR	NR	NR	NR
	Accuracy speed ratio	3.54	76.0	79.0	0.84 (NR)	NR	NR	NR	NR
Dwolatzky et al^34^	Mindstreams computerised cognitive testing	NA for AUC	NR	NR	0.84 (NR)	NR	NR	NR	NR
Kingsbury et al^37^	CogniScreen								
	Pair recognition	0.47	76.0	60.0	0.72 (0.62‐0.83)	NR	NR	NR	NR
	Cued recall	0.305	82.1	76.7	0.87 (0.80‐0.95)	NR	NR	NR	NR
	Immediate and delayed serial recall	0.385	92.6	80.0	0.89 (0.81‐0.97)	NR	NR	NR	NR
Kluger et al^38^	Computerised test (no name)	NR	NR	NR	0.89	NR	NR	NR	NR
Maruff et al^40^	CBB								
	Psychomotor/attention	90	41.1	85.7	0.67 (0.6‐0.73)	NR	NR	NR	NR
	Learning/working memory	90	80.4	84.7	0.91 (0.87‐0.94)	NR	NR	NR	NR
Tierney et al^45^	CAMCI	2	80.0	74.0	NR	NR	NR	NR	NR
Vacante et al^46^	Computerised total (novel and traditional)	19.5	70.0	76.2	NR	NR	NR	NR	NR
	Computerised objects and faces (novel and traditional)	12.5	50	64.3	NR	NR	NR	NR	NR
	Computerised objects and faces (novel and traditional)	13.5	75	52.4	NR	NR	NR	NR	NR
Early dementia									
Doniger et al^33^	Mindstreams (abridged)								
	Overall	NA	NR	NR	0.886	NR	NR	NR	NR
	Memory								
	Verbal memory		NR	NR	0.830 (0.762‐0.898)	NR	NR	NR	NR
	Nonverbal memory		NR	NR	0.825 (0.756‐0.893)	NR	NR	NR	NR
	Executive function								
	Go–No Go		NR	NR	0.733 (0.640‐0.826)	NR	NR	NR	NR
	Stoop interference		NR	NR	0.790 (0.690‐0.890)	NR	NR	NR	NR
	Catch game		NR	NR	0.748 (0.670‐0.827)	NR	NR	NR	NR
	Visual spatial								
	Visual spatial imagery		NR	NR	0.678 (0.567‐0.789)	NR	NR	NR	NR
Dwolatzky et al^34^	Mindstreams computerized cognitive testing	NR	NR	NR	NR	NR	NR	NR	NR
Kluger et al^38^	Computerised test (no name)	NR	NR	NR	0.97	NR	NR	NR	NR
Vacante et al^46^	TPT								
	Computerised total (novel and traditional)	15.5	88.9	92.9	NR	NR	NR	NR	NR
	Computerised objects and faces (novel and traditional)	11.5	94.4	78.6	NR	NR	NR	NR	NR
	Computerised objects and faces (novel and traditional)	13.5	94.4	52.4	NR	NR	NR	NR	NR
MCI/early dementia									
Doniger et al^33^	Mindstreams (abridged)								
	Overall	NA for AUC	NR	NR	0.823 (0.757‐0.888)	NR	NR	NR	NR
	Memory								
	Verbal memory				0.773(0.697‐0.849)				
	Nonverbal memory				0.767 (0.690‐0.844)				
	Executive function								
	Go–No Go				0.719 (0.639‐0.800)				
	Stoop interference				0.671 (0.575‐0.766)				
	Catch game				0.685 (0.595‐0.776)				
	Visual spatial								
	Visual spatial imagery				0.721 (0.638‐0.803)				
Lichtenberg et al^39^	CST	1.5	80.0	87.0	NR	88.0	79.0	NR	NR

Abbreviations: AUC, area under curve; CAMCI, Computer Assessment of Mild Cognitive Impairment; CANS‐MCI, Computer‐Administered Neuropsychological Screen for Mild Cognitive Impairment; CBB, CogState Brief Battery; CST, Computerised Self‐Test; LR−, negative likelihood ratio; LR+, positive likelihood ratio; MCI, mild cognitive impairment; NA, not applicable; NPV, negative predictive value; NR, not reported; PPV, positive predictive value; TPT, The Placing Test.

#### 3.3.1 Mild cognitive impairment

Eight studies reported the diagnostic accuracy outcomes for MCI. Ahmed et al evaluated Computer‐Administered Neuropsychological Screen for Mild Cognitive Impairment and reported high sensitivity (89.0%) and moderate specificity (73.0%) with an AUC of 0.867, which shows a good ability to discriminate between the MCI group and the non‐MCI group. Tierney et al evaluated the CAMCI test and reported a high sensitivity (80.0%) and a moderate specificity (74.0%); the authors did not report AUC values. Maruff et al evaluated the CogState Brief Battery (CBB). The CogState Brief Battery has 2 composite scores for 4 tasks: psychomotor function, attention function, learning memory, and working memory. The psychomotor/attention function had poor discrimination since its AUC was 0.67. It also had poor sensitivity (41.1%) but high specificity (85.7%). The AUC for the learning/working memory was 0.91, which shows exceptional ability to discriminate between the MCI group and the non‐MCI group. It also had high sensitivity (80.4%) and high specificity (84.7%). The overall sensitivity, specificity, and AUC were not reported.

#### 3.3.2 Early dementia

Dwolatzky et al^34^ and Doniger et al^33^ both assessed the Mindstreams computerised cognitive testing. Only Doniger et al reported results relating to early dementia. They evaluated an abridged version of Mindstreams with an overall AUC of 0.886, which showed a good ability to discriminate between the early‐dementia group and the non–early‐dementia group.

#### 3.3.3 MCI/early dementia

Kluger et al evaluated an automated computerised test, which did not have a specific name. The authors reported an AUC of 0.97, which shows exceptional ability to discriminate between early dementia and healthy controls.

Doniger et al reported an overall AUC of 0.823, which showed a good ability to discriminate between the cognitively healthy group and the cognitive unhealthy group. The AUC values for individual test results ranged from 0.671 to 0.773.

Lichtenberg et al^39^ reported sensitivity and specificity values (80.0% and 87.0%, respectively), PPV (88.0%), and NPV (79.0%).

#### 3.3.4 HIV‐associated neurocognitive disorders

One study^43^ evaluated diagnostic accuracy of an automated computerised test that included people with cognitive impairment with co‐morbidities. This study examined the HAND and used the automated test CAMCI. The CAMCI test assessed multiple domains with different tasks. The study examined a range of diagnostic accuracy outcomes but did not report the values for all of them.

#### 3.3.5 Methodological quality

**Figure 2:**
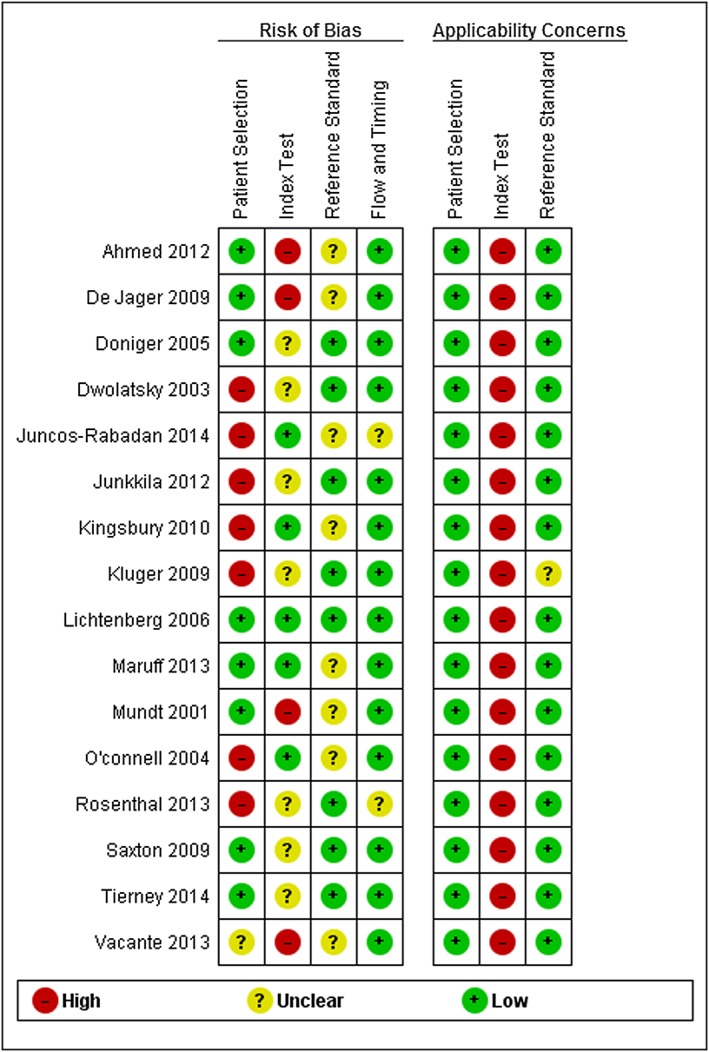
Risk of bias and applicability concerns summary [Colour figure can be viewed at wileyonlinelibrary.com]

The methodological quality of the included studies was assessed using the Quality Assessment of Diagnostic Accuracy Studies tool as summarised in Figure 2.

The risk‐of‐bias criterion for patient selection was high for 7 studies because a case‐control study design had not been avoided (see Appendix S6 found in the Supporting Information). Seven studies were judged to be at unclear risk in the index test criteria for risk of bias since the threshold values for the index tests were not prespecified. There was high concern regarding the applicability of the index test for all of the studies because the interpretation of the index test was different from the review question, since it is not possible to establish diagnosis of MCI and early dementia using automated computerised tests in isolation; specialist expertise is necessary to establish a diagnosis.

The reference standard domain for the risk of bias was unclear in 8 studies since it was not possible to ascertain whether reference standard results were interpreted without knowledge of the results of the index tests. All but one study^38^ were judged to have low concern for applicability regarding the reference standard since it used a consensus of 2 clinicians' opinions as the reference standard. In the flow and timing domain for the risk of bias, a judgement of unclear risk of bias was given to 2 studies^35^, ^43^ since attrition or timing was not described in the papers. However, 14 studies were assessed as being at low risk because all patients had received the same reference standard and all patients were included in the analysis. There was a high concern in the domains of applicability for 16 studies. Of the 16 studies, only 1 was judged to be at low of risk of bias across the 4 domains examined^39^; despite this, the overall quality of the included studies was considered to be good.

### 3.4 Patient and public involvement

Data from the included studies were presented and discussed with a service user. The structure of the meeting is described in Appendix S5 found in the Supporting Information. The service user thought that all of the index text domains needed to be tested to enable a comprehensive overview of any suspected cognitive impairment. His view was that more information on key domains would help clinicians and patients address the challenges faced by patients with MCI or early dementia. The service user raised concerns about the age of the study participants since there were no tests that assessed cognitive impairment in people over the age of 90 years. Another concern was the effect of little or no education on the ability to perform well on the test. The importance of the index tests being user‐friendly and acceptable to patients was also highlighted. He also stated a preference for desktop computers over touch screen test, in case a patient had tremors. He also highlighted the importance of ensuring that the colour palette in visual components of the tests had a sharp contrast because it is likely that older people will have problems with their eyesight. He also stated that some people might become frustrated with tests that lasted longer than 40 minutes.

## 4 DISCUSSION

In assessing the diagnostic accuracy of a test, an index test with high specificity is preferable for diagnosis, and high sensitivity is preferred for screening.^47^ When patients are diagnosed with MCI or early dementia, an index test with both high sensitivity and specificity is needed to be able to appreciate a distinctive pattern of cognitive impairment in MCI and early dementia. This distinctive pattern of cognitive impairment distinguishes the cognitive impairment caused by another disease process, eg, cognitive impairment as presented in depression or HIV.

A number of studies included in this review were not conducted in samples representative of the usual clinical population in which these tests might be used (eg, patients visiting the memory clinics with a mix of MCI and dementia of various aetiologies and the “worried well” and depressed patients) but were conducted in convenience samples of patients with limited diagnoses (mostly MCI and AD). This, along with the lack of reliable evidence to support one test over the other, makes it difficult to draw a clear picture of the diagnostic accuracy of the index tests in this review.

There was some disparity in how the studies were reported; for example, all of the index tests, except 4, were used as screening tests, yet the authors reported outcomes for diagnostic accuracy. It is also not clear from reviewing the included studies whether these computerised tests ought to be used in primary or secondary care. In the United Kingdom, some primary care practices take part in “case finding” for dementia, for example, targeting “high‐risk” groups (eg, older adults or patients with high vascular risk, learning disability, or Parkinson disease), and hospital staff undertake brief cognitive assessments during all acute admissions for older adults.

The pen‐and‐paper tests currently used in clinical practice not only help clinicians differentiate between normal cognition, MCI, and dementia^20^, ^21^, ^22^ but also assist in staging severity of illness. The CANTAB test was the only automated test that could stage severity.^35^, ^36^, ^42^ But 2 of the 3 CANTAB‐PAL studies^36^, ^42^ had very small sample sizes (58 and 50, respectively), and the slightly larger study^35^ only tested the domain of visual episodic memory. The time taken to complete these computerised tests is not clear in the case of CANTAB‐PAL and depending on the version of Mindstreams, ranged from 30 to 45 minutes.^33^ In contrast, the paper‐based tests range from 7 to 10 minutes in their application.^20^, ^21^, ^22^ Concern for the time it takes to complete the tests was raised in the service user feedback; the user pointed out the possibility of people becoming frustrated with tests that lasted for more than 40 minutes, especially if they are not familiar with using technology. The data in the included papers also did not describe the time needed for training the assessor and the need for a specialist for scoring.

An important point to consider is that current diagnosis of patients with MCI and early dementia is based on clinical judgement and medical history as well as on the results of paper‐based cognitive tests. Automated tests cannot be used in isolation or substituted for clinical judgement. Even with prespecified cut‐off values for a particular population, any cognitive testing measure alone is insufficient to render a diagnostic classification.

None of the previously conducted relevant reviews in this area conducted a diagnostic accuracy review.^23^, ^48^, ^49^ They were narrative reviews that provided a summary of the battery of tests used and rated this evidence on validity and reliability, comprehensiveness, and usability. This review focused on computerised tests that were self‐administered and had a minimum level of involvement from professionals. In line with the findings of this review, the authors of the other reviews concluded that there is significant difference in automated computerised tests, and hence, they must be judged on a case‐by‐case basis.^23^

More research is required to establish stable cut‐off points for each automated test used to diagnose patients with MCI or early dementia. An important consideration is testing the cut‐off points in specific patient populations, for example, in patients of different age groups or education levels and from different geographical regions.

Another area for future research is providing more information on the costs of automated tests and include time for training, administration, and scoring of the different tests, as these are important factors for their use in routine clinical practice. This information is currently absent in the published studies describing automated tests used to diagnose or monitor people with MCI or early dementia. No studies reporting on outcomes relating to monitoring progression of disease could be identified, which highlights a difficulty in the current method of monitoring progression and treatment response compared with standard clinical practice.

### 4.1 Strengths of this review

The search strategy for this review was extensive. The methodological rigour of the review process was enhanced by the use of 2 assessors to perform citation screening, quality assessment, and data extraction/checking. All of the primary study authors were contacted and asked to fill in the contingency tables. A patient and public involvement exercise was also conducted.

### 4.2 Weaknesses of the review

This review is limited in part by the number of included studies for the same automated computerised test. Because of noncomparable data relating to the index test, it was not appropriate to pool the data. Another limitation with the studies is the lack of comparative results across the different domains being examined.

## 5 CONCLUSIONS

It is difficult to draw a clear picture of the diagnostic accuracy of automated computerised tests to establish a diagnosis of MCI or early dementia in this review because there is currently insufficient evidence to support the use of one test over the other. Further research is required to examine the cut‐off points for the diagnosis of MCI and early dementia when using automated tests. These test scores do not always relate with medical history and more importantly with functioning. The suitability of these tests also depends on their cost, time needed for training the assessor, time needed for the administration of the test, and the need for a specialist for scoring.

## CONFLICT OF INTEREST

None declared.

## FUNDING

The NIHR Health Technology Assessment Programme commissioned this report with project reference number 15/67/01.

## Supporting information


**Data S1**.
Supporting information
