# Supplementary material for: A systematic review of the diagnostic accuracy of automated tests for cognitive impairment
Source: Int J Geriatr Psychiatry. 2018 Jan 22;33(4):561–75. doi: 10.1002/gps.4852 (PMC5887872; doi:10.1002/gps.4852)
Supplement: Supplementary file 1 — Data S1. Supporting information [file GPS-33-561-s001.docx]

## Appendix 1 Abbreviations list

| AD | Alzheimer’s disease |
| --- | --- |
| ADL | Activities of daily living |
| ANAM | Automated neuropsychological assessment matrix |
| AUC | Area under the curve |
| CAMCI | Computer assessment of mild cognitive impairment |
| CANS-MCI | Computer-administered neuropsychological screen for mild cognitive impairment |
| CANTAB | Cambridge neuropsychological test automated battery |
| CANTAB-PAL | Cambridge neuropsychological test automated battery paired association learning |
| CBB | CogState brief battery |
| CI | Confidence interval |
| Cogdras | Cognitive drug research computerised assessment system |
| CSI | Community screening interview |
| CST | Computerised self-test |
| DMS | Delayed matching to sample |
| DSM IV, V | Diagnostic and Statistical Manual of Mental Disorders IV, V |
| FPR | False positive rate |
| GP | General Practitioner |
| GPCOG | General Practitioner assessment of cognition |
| HIV | Human immunodeficiency virus |
| HAD | HIV-associated dementia |
| HAND | HIV-associated neurocognitive disorders |
| HVLT | Hopkins verbal learning test |
| ICD-10 | International Classification of Disease |
| LR | Likelihood ratio |
| MAS | Memory Assessment Service |
| MCI | Mild cognitive impairment |
| MCIS | Mild cognitive impairment screen |
| MMSE | Mini-mental state examination |
| NINCDS-ADRDA | National Institute of Neurological and Communicative Disorders and Stroke and the Alzheimer's Disease and Related Disorders Association |
| NPV | Negative predictive value |
| PAL | Paired associated learning |
| PPV | Positive predictive value |
| PRM | Pattern recognition memory |
| QUADAS | Quality Assessment Tool for Diagnostic Accuracy Studies |
| RCT | Randomised controlled trial |
| ROC | Receiver operating characteristic |
| SAS | Statistical Analysis Software |
| SROC | Summary receiver operating characteristic |
| TPR | True positive rate |
| TPT | The placing test |

## Appendix 2 Search strategy conducted in Medline

|  | Search terms | Results |
| --- | --- | --- |
| 1 | exp mild cognitive impairment/ | 3249 |
| 2 | ((early* or onset* or initial* or young* or incipient*) adj2 (dementia* or Alzheimer* or AD)).tw. | 9646 |
| 3 | ((Mild* or early* or onset* or initial* or progress* or minor or young* or moderat* or suspect*) adj2 Cognit* adj1 (impair* or disord* or diseas* or declin* or deteriorat* or fail* or complain* or dysfunct* or degenerat* or deficit*)).tw. | 11933 |
| 4 | MCI.tw. | 11603 |
| 5 | NCD.tw. | 1233 |
| 6 | ((memory* or neurocognitiv*) adj2 (impair* or disord* or diseas* or declin* or deteriorat* or fail* or complain* or dysfunct* or degenerat* or deficit*)).tw. | 29085 |
| 7 | ("preclinical alzheimer*" or "pre-clinical alzheimer*").tw. | 293 |
| 8 | (prodrom* adj2 dement*).tw. | 84 |
| 9 | *dementia/ or *alzheimer disease/ or *dementia, vascular/ or *dementia, multi-infarct/ or *frontotemporal dementia/ | 88482 |
| 10 | or/1-9 | 129676 |
| 11 | ((computer* or automate*) adj2 (test* or assess* or evaluat* or screen* or battery or monitor* or identif* or assess* or evaluat* or interpret*)).tw. | 19607 |
| 12 | (automat* adj2 (interpretat* or test*)).tw. | 2445 |
| 13 | *Neuropsychological Tests/ | 13160 |
| 14 | ((neuropsychological or neuro-psychological or psychometric*) adj5 (computer* or automate*) adj5 (test* or assess* or evaluat* or screen* or battery or monitor* or identif* or assess* or evaluat* or interpret*)).tw. | 831 |
| 15 | psychometrics/ | 58160 |
| 16 | or/11-15 | 87870 |
| 17 | 10 and 16 | 5551 |
| 18 | Diagnosis, Computer-Assisted/ | 19850 |
| 19 | ((computer* or automate*) adj4 (diagnos* or detect*)).tw. | 13645 |
| 20 | or/18-19 | 30836 |
| 21 | 10 and 20 | 366 |
| 22 | disease progression/ | 114210 |
| 23 | ((test* or assess* or evaluat* or screen* or battery or monitor* or identif* or assess* or evaluat* or interpret*) adj3 diseas* adj3 (progress* or exacerbat*)).tw. | 4249 |
| 24 | or/22-23 | 117390 |
| 25 | 17 and 24 | 342 |
| 26 | "cambridge Neuropsychology Test*".tw. | 0 |
| 27 | Computerised Neuropsychological Test Battery.tw. | 38 |
| 28 | Six Item Cognitive Impairment Test.tw. | 11 |
| 29 | "Computer Assessment of Mild Cognitive Impairment".tw. | 3 |
| 30 | MindStream*.tw. | 27 |
| 31 | "Mild Cognitive Impairment Screen*".tw. | 11 |
| 32 | Computer Administered Neuropsychological Screen for Mild Cognitive Impairment.tw. | 2 |
| 33 | Automated Neuropsychological Assessment Metrics.tw. | 90 |
| 34 | CANS-MCI.tw. | 2 |
| 35 | "CNS Vital Signs".tw. | 33 |
| 36 | Cognitive Drug Research Computerised Assessment System for Dementia.tw. | 1 |
| 37 | CogState.tw. | 93 |
| 38 | "Cognitive Stability Index*".tw. | 6 |
| 39 | "Cognitive Screening Test*".tw. | 183 |
| 40 | Microcog.tw. | 28 |
| 41 | (COGDRAS-D or COGDRASD or COGDRAS).tw. | 0 |
| 42 | MCIS.tw. | 167 |
| 43 | (CAMCI or CNTB).tw. | 20 |
| 44 | 6CIT.tw. | 11 |
| 45 | (CANTAB-A or CANTABA or CANTAB).tw. | 343 |
| 46 | ANAM.tw. | 128 |
| 47 | CADi2.tw. | 1 |
| 48 | or/26-46 | 1103 |
| 49 | 17 or 21 or 25 or 48 | 6733 |
| 50 | animals/ not humans/ | 3996470 |
| 51 | 49 not 50 | 6691 |
| 52 | comment/ or editorial/ or letter/ | 1496207 |
| 53 | case reports/ | 1764849 |
| 54 | (comment or editorial or letter or journal correspondence or opinion).pt. | 1496207 |
| 55 | or/52-54 | 3069853 |
| 56 | 51 not 55 | 6360 |
| 57 | limit 56 to yr="2005 -Current" | 3779 |

## Appendix 3 Number of studies retrieved from different databases

| Databases | Date searched | Version/files | No. retrieved |
| --- | --- | --- | --- |
| Cochrane Database of Systematic Reviews – CDSR (Cochrane) | 06/08/2015 | Issue 7 of 12, July 2015 | 684 |
| Database of Abstracts of Reviews of Effects – DARE (Cochrane) | 06/08/2015 | Issue 7 of 12, July 2015 | 32 |
| HTA database (Cochrane) | 06/08/2015 | Issue 7 of 12, July 2015 | 4 |
| Cochrane Central Database of Controlled Trials – CENTRAL (Cochrane) | 06/08/2015 | Issue 7 of 12, July 2015 | 1136 |
| NHS EED | 06/08/2015 | Issue 7 of 12, July 2015 | 7 |
| MEDLINE (Ovid) and MEDLINE In-Process (Ovid) | 06/08/2015 | Ovid MEDLINE(R) 1946 to July Week 5 2015, Database Field Guide Ovid MEDLINE(R) In-Process & Other Non-Indexed Citations August 05, 2015 | 3779 |
| EMBASE (Ovid) | 06/08/2015 | 1980 to 2015 Week 31 | 3191 |
| Web of Science | 06/08/2015 | N/A | 3586 |
| Psychinfo | 06/08/2015 | N/A | 541 |
| Pubmed | 03/09/2015 | N/A | 392 |
| Proquest theses and dissertations | 03/09/2015 | N/A | 197 (main search)  1919 (search for test names) |
| Handsearching | 26/08/2015 | N/A | 4 |
| Citation tracking | 19/09/2015 | N/A | 3324 |

## Appendix 4 Handsearching for trials

| Databases | Date searched | Links to results |
| --- | --- | --- |
| Clinicaltrials.gov | 26/08/2015 | [A Study of a Self-Administered Memory Screening Test With Automated Reporting (SAMSTAR) in Participants With Mild Cognitive Impairment](https://clinicaltrials.gov/ct2/show/NCT02419183?term=computer+tests+MCI&rank=1){Janssen Research & Development, 2015 #1578}  [Pilot Testing a New Computer-based Screening Tool to Detect Cognitive Impairment](https://clinicaltrials.gov/ct2/show/NCT01220674?term=computer+tests+MCI&rank=2){Singer, 2011 #1579}  [Feasibility and Validity of A Novel Computer Based Battery of Assessments in the Elderly (HHT)](https://clinicaltrials.gov/show/NCT02109419){CK, 2014 #1580}  [Computational Tools for Early Diagnosis of Memory Disorders (ProsKuopio)](https://clinicaltrials.gov/show/NCT02050464){Soininen, 2014 #1581;Soininen, 2014 #1581} |
| metaRegister of Controlled Trials and ISRCTN Register | 26/08/2015 | None found |
| WHO International Clinical Trials Registry Platform | 26/08/2015 | None found |
| Prospero systematic review register | 26/08/2015 | None found |
| Epistemonikos | 26/08/2015 | None new references found |

## Appendix 5 PPI involvement - structure of meeting

Brief chat, any difficulties with train trip, any questions he may have. Check for comfort with recording device, assure recording will be deleted. If not, use tablet for quick typing. He mentioned his caring responsibilities. Talk slowly, pay attention to ability to retain and analyse information. Simple language.

What is LRiG. Overview of the project. Brief discussion background MCI and dementia. Explain the review process very briefly.

Intro questions from protocol

- Was the purpose of the review clear?
- Was the purpose of the review appropriate/valid/important?

Questions relating to technology

Explain what computerised tests are briefly. Compare to tests used in his and/or carers diagnosis

1. About the tests (Show table on test, discuss table, clarify, make sure its understood)

- Different types of tests (show table), some tests look at 1-2 aspects of memory and thinking, others look at all aspects of memory and thinking (cognitive domains).
- 2 tests look at the visual aspect (PAL), and make an assessment with a clinician present about existence of challenges with memory and thinking
- Our findings say it works well. Does he have any thoughts about this?

1. Platform used

- Internet based test, iPad vs computer touch screen vs desktop. Rating 1-4
- Comfort in using platform (create flow from previous questions so would talk about use of technology in the elderly

1. Professionals involved

- Different papers used professionals differently.
- For test administration it was 1) self-administered with minimum assistance with nurse or technician 2) self-administered with minimum assistance with nurse or technician, which also included them typing or using the mouse to manipulate the mouse or arrows for the patient or facilitate the typing and entering of patient responses. 3) Verbal directions from administration 4) interactive system provided oral instruction through speakers and written instructions on screen 5) self-administration, but carer can assist needed them typing or using the mouse to manipulate the mouse or arrows for the patient or facilitate the typing and entering of patient responses
- Rate tests from 1-5 with 1 being most preferable. Enter sixth option manually if a combination
- Feedback on professionals conducting the test (who)
- What did he think of the level of involvement of the assessor in these scenarios in conducting the tests?
- what about monitoring

1. Patient characteristics

- disease progression, do they go to the doctor,
- follow-up
- study sample
- where people were recruited from
- only included patients (summary and variance of inclusion criteria)
- summary of age, gender, education, ethnicity (race)

Follow-up questions in the end

- Do we report on all of the important aspects of the research (show tables? e.g. age of participants, practicality of computer tests for this age group?
- Was the purpose of the review clear?
- Was the purpose of the review appropriate/valid/important?
- What do they see as the implications for clinical practice?
- What value do you see in the use of diagnostic tests for MCI?
- Do you feel that the inclusion of computerised tests is a good one?
- Should they be used instead of pen-and-paper tests?
- When do you think they should be used?
- What areas need further research?

## Appendix 6 Risk of bias assessment (detail)

**Ahmed 2012**

**Patient Selection**

| **A. Risk of Bias** | | |
| --- | --- | --- |
| Patient Sampling |  | |
| Was a consecutive or random sample of patients enrolled? | |  |
| Was a case-control design avoided? | |  |
| Did the study avoid inappropriate exclusions? | |  |
| **Could the selection of patients have introduced bias?** | |  |

| **B. Concerns regarding applicability** | | |
| --- | --- | --- |
| Patient characteristics and setting |  | |
| **Are there concerns that the included patients and setting do not match the review question?** | |  |

**Index Test**

| Index tests | CANS-MCI |
| --- | --- |

**All tests**

| **A. Risk of Bias** | |
| --- | --- |
| Were the index test results interpreted without knowledge of the results of the reference standard? |  |
| If a threshold was used, was it pre-specified? |  |
| **Could the conduct or interpretation of the index test have introduced bias?** |  |

| **B. Concerns regarding applicability** | |
| --- | --- |
| **Are there concerns that the index test, its conduct, or interpretation differ from the review question?** |  |

**Reference Standard**

| **A. Risk of Bias** | | |
| --- | --- | --- |
| Target condition and reference standard(s) | Clinical diagnosis using Petersen's criteria | |
| Is the reference standards likely to correctly classify the target condition? | |  |
| Were the reference standard results interpreted without knowledge of the results of the index tests? | |  |
| **Could the reference standard, its conduct, or its interpretation have introduced bias?** | |  |

| **B. Concerns regarding applicability** | |
| --- | --- |
| **Are there concerns that the target condition as defined by the reference standard does not match the question?** |  |

**Flow and Timing**

| **A. Risk of Bias** | | |
| --- | --- | --- |
| Flow and timing | 70 mins of testing. Not stated time between reference. | |
| Was there an appropriate interval between index test and reference standard? | |  |
| Did all patients receive the same reference standard? | |  |
| Were all patients included in the analysis? | |  |
| **Could the patient flow have introduced bias?** | |  |

**Notes**

| **Notes** | Reference not explained in paper. |
| --- | --- |

**de Jager 2009**

**Patient Selection**

| **A. Risk of Bias** | | |
| --- | --- | --- |
| Patient Sampling | Consecutive | |
| Was a consecutive or random sample of patients enrolled? | |  |
| Was a case-control design avoided? | |  |
| Did the study avoid inappropriate exclusions? | |  |
| **Could the selection of patients have introduced bias?** | |  |

| **B. Concerns regarding applicability** | | |
| --- | --- | --- |
| Patient characteristics and setting | Healthy, community-dwelling adults aged ≥60 without significantprogressive, subjective memory complaints. Primary Care setting | |
| **Are there concerns that the included patients and setting do not match the review question?** | |  |

**Index Test**

| Index tests | CogState |
| --- | --- |

**All tests**

| **A. Risk of Bias** | |
| --- | --- |
| Were the index test results interpreted without knowledge of the results of the reference standard? |  |
| If a threshold was used, was it pre-specified? |  |
| **Could the conduct or interpretation of the index test have introduced bias?** |  |

| **B. Concerns regarding applicability** | |
| --- | --- |
| **Are there concerns that the index test, its conduct, or interpretation differ from the review question?** |  |

**Reference Standard**

| **A. Risk of Bias** | | |
| --- | --- | --- |
| Target condition and reference standard(s) | Clinical diagnosis using battery of neurocognitive tests | |
| Is the reference standards likely to correctly classify the target condition? | |  |
| Were the reference standard results interpreted without knowledge of the results of the index tests? | |  |
| **Could the reference standard, its conduct, or its interpretation have introduced bias?** | |  |

| **B. Concerns regarding applicability** | |
| --- | --- |
| **Are there concerns that the target condition as defined by the reference standard does not match the question?** |  |

**Flow and Timing**

| **A. Risk of Bias** | | |
| --- | --- | --- |
| Flow and timing | 1-year after reference test. | |
| Was there an appropriate interval between index test and reference standard? | |  |
| Did all patients receive the same reference standard? | |  |
| Were all patients included in the analysis? | |  |
| **Could the patient flow have introduced bias?** | |  |

**Notes**

| **Notes** |  |
| --- | --- |

**Doniger 2005**

**Patient Selection**

| **A. Risk of Bias** | | |
| --- | --- | --- |
| Patient Sampling | elderly individuals who attended one of three tertiary care memory clinics and an assisted living facility | |
| Was a consecutive or random sample of patients enrolled? | |  |
| Was a case-control design avoided? | |  |
| Did the study avoid inappropriate exclusions? | |  |
| **Could the selection of patients have introduced bias?** | |  |

| **B. Concerns regarding applicability** | | |
| --- | --- | --- |
| Patient characteristics and setting |  | |
| **Are there concerns that the included patients and setting do not match the review question?** | |  |

**Index Test**

| Index tests | Mindstreams |
| --- | --- |

**All tests**

| **A. Risk of Bias** | |
| --- | --- |
| Were the index test results interpreted without knowledge of the results of the reference standard? |  |
| If a threshold was used, was it pre-specified? |  |
| **Could the conduct or interpretation of the index test have introduced bias?** |  |

| **B. Concerns regarding applicability** | |
| --- | --- |
| **Are there concerns that the index test, its conduct, or interpretation differ from the review question?** |  |

**Reference Standard**

| **A. Risk of Bias** | | |
| --- | --- | --- |
| Target condition and reference standard(s) |  | |
| Is the reference standards likely to correctly classify the target condition? | |  |
| Were the reference standard results interpreted without knowledge of the results of the index tests? | |  |
| **Could the reference standard, its conduct, or its interpretation have introduced bias?** | |  |

| **B. Concerns regarding applicability** | |
| --- | --- |
| **Are there concerns that the target condition as defined by the reference standard does not match the question?** |  |

**Flow and Timing**

| **A. Risk of Bias** | | |
| --- | --- | --- |
| Flow and timing |  | |
| Was there an appropriate interval between index test and reference standard? | |  |
| Did all patients receive the same reference standard? | |  |
| Were all patients included in the analysis? | |  |
| **Could the patient flow have introduced bias?** | |  |

**Notes**

| **Notes** |  |
| --- | --- |

**Dwolatsky 2003**

**Patient Selection**

| **A. Risk of Bias** | | |
| --- | --- | --- |
| Patient Sampling | assessed and selected at primary care clinics | |
| Was a consecutive or random sample of patients enrolled? | |  |
| Was a case-control design avoided? | |  |
| Did the study avoid inappropriate exclusions? | |  |
| **Could the selection of patients have introduced bias?** | |  |

| **B. Concerns regarding applicability** | | |
| --- | --- | --- |
| Patient characteristics and setting | Not a UK study | |
| **Are there concerns that the included patients and setting do not match the review question?** | |  |

**Index Test**

| Index tests | Mindstreams |
| --- | --- |

**All tests**

| **A. Risk of Bias** | |
| --- | --- |
| Were the index test results interpreted without knowledge of the results of the reference standard? |  |
| If a threshold was used, was it pre-specified? |  |
| **Could the conduct or interpretation of the index test have introduced bias?** |  |

| **B. Concerns regarding applicability** | |
| --- | --- |
| **Are there concerns that the index test, its conduct, or interpretation differ from the review question?** |  |

**Reference Standard**

| **A. Risk of Bias** | | |
| --- | --- | --- |
| Target condition and reference standard(s) | Clinical diagnosis battery of neurocognitive tests | |
| Is the reference standards likely to correctly classify the target condition? | |  |
| Were the reference standard results interpreted without knowledge of the results of the index tests? | |  |
| **Could the reference standard, its conduct, or its interpretation have introduced bias?** | |  |

| **B. Concerns regarding applicability** | |
| --- | --- |
| **Are there concerns that the target condition as defined by the reference standard does not match the question?** |  |

**Flow and Timing**

| **A. Risk of Bias** | | |
| --- | --- | --- |
| Flow and timing | not recorded | |
| Was there an appropriate interval between index test and reference standard? | |  |
| Did all patients receive the same reference standard? | |  |
| Were all patients included in the analysis? | |  |
| **Could the patient flow have introduced bias?** | |  |

**Notes**

| **Notes** | Assessors were blind to results except for McGill Jewish hospital where they were aware of diagnosis but were not involved in determination of results |
| --- | --- |

**Juncos-Rabadan 2014**

**Patient Selection**

| **A. Risk of Bias** | | |
| --- | --- | --- |
| Patient Sampling | Exptal: recruited from participants who took part in the ongoing longitudinal cognitive assessment studies  Controls: Participants scoring higher than the cut-off on memory, general cognitive functioning, and specific cognitive domain tests were recruited from the same Primary Care Health Centres | |
| Was a consecutive or random sample of patients enrolled? | |  |
| Was a case-control design avoided? | |  |
| Did the study avoid inappropriate exclusions? | |  |
| **Could the selection of patients have introduced bias?** | |  |

| **B. Concerns regarding applicability** | | |
| --- | --- | --- |
| Patient characteristics and setting |  | |
| **Are there concerns that the included patients and setting do not match the review question?** | |  |

**Index Test**

| Index tests | CANTAB |
| --- | --- |

**All tests**

| **A. Risk of Bias** | |
| --- | --- |
| Were the index test results interpreted without knowledge of the results of the reference standard? |  |
| If a threshold was used, was it pre-specified? |  |
| **Could the conduct or interpretation of the index test have introduced bias?** |  |

| **B. Concerns regarding applicability** | |
| --- | --- |
| **Are there concerns that the index test, its conduct, or interpretation differ from the review question?** |  |

**Reference Standard**

| **A. Risk of Bias** | | |
| --- | --- | --- |
| Target condition and reference standard(s) | Clinical diagnosis using neurocognitive tests, NINCDS-ADRDA and DMS-IV criteria | |
| Is the reference standards likely to correctly classify the target condition? | |  |
| Were the reference standard results interpreted without knowledge of the results of the index tests? | |  |
| **Could the reference standard, its conduct, or its interpretation have introduced bias?** | |  |

| **B. Concerns regarding applicability** | |
| --- | --- |
| **Are there concerns that the target condition as defined by the reference standard does not match the question?** |  |

**Flow and Timing**

| **A. Risk of Bias** | | |
| --- | --- | --- |
| Flow and timing | No details of timing or attrition | |
| Was there an appropriate interval between index test and reference standard? | |  |
| Did all patients receive the same reference standard? | |  |
| Were all patients included in the analysis? | |  |
| **Could the patient flow have introduced bias?** | |  |

**Notes**

| **Notes** |  |
| --- | --- |

**Junkkila 2012**

**Patient Selection**

| **A. Risk of Bias** | | |
| --- | --- | --- |
| Patient Sampling | aMCI and AD pts. consecutive, Controls recruited through community | |
| Was a consecutive or random sample of patients enrolled? | |  |
| Was a case-control design avoided? | |  |
| Did the study avoid inappropriate exclusions? | |  |
| **Could the selection of patients have introduced bias?** | |  |

| **B. Concerns regarding applicability** | | |
| --- | --- | --- |
| Patient characteristics and setting | Controls were from the community. 6 Pts with probable AD were receiving medication for AD | |
| **Are there concerns that the included patients and setting do not match the review question?** | |  |

**Index Test**

| Index tests | CANTAB-PAL |
| --- | --- |

**All tests**

| **A. Risk of Bias** | |
| --- | --- |
| Were the index test results interpreted without knowledge of the results of the reference standard? |  |
| If a threshold was used, was it pre-specified? |  |
| **Could the conduct or interpretation of the index test have introduced bias?** |  |

| **B. Concerns regarding applicability** | |
| --- | --- |
| **Are there concerns that the index test, its conduct, or interpretation differ from the review question?** |  |

**Reference Standard**

| **A. Risk of Bias** | | |
| --- | --- | --- |
| Target condition and reference standard(s) | Clinical diagnosis using Petersen's criteria and neurocognitive tests | |
| Is the reference standards likely to correctly classify the target condition? | |  |
| Were the reference standard results interpreted without knowledge of the results of the index tests? | |  |
| **Could the reference standard, its conduct, or its interpretation have introduced bias?** | |  |

| **B. Concerns regarding applicability** | |
| --- | --- |
| **Are there concerns that the target condition as defined by the reference standard does not match the question?** |  |

**Flow and Timing**

| **A. Risk of Bias** | | |
| --- | --- | --- |
| Flow and timing | 20% attrition reasons stated. No details on timing | |
| Was there an appropriate interval between index test and reference standard? | |  |
| Did all patients receive the same reference standard? | |  |
| Were all patients included in the analysis? | |  |
| **Could the patient flow have introduced bias?** | |  |

**Notes**

| **Notes** |  |
| --- | --- |

**Kingsbury 2010**

**Patient Selection**

| **A. Risk of Bias** | | |
| --- | --- | --- |
| Patient Sampling |  | |
| Was a consecutive or random sample of patients enrolled? | |  |
| Was a case-control design avoided? | |  |
| Did the study avoid inappropriate exclusions? | |  |
| **Could the selection of patients have introduced bias?** | |  |

| **B. Concerns regarding applicability** | | |
| --- | --- | --- |
| Patient characteristics and setting |  | |
| **Are there concerns that the included patients and setting do not match the review question?** | |  |

**Index Test**

| Index tests | CogniScreen |
| --- | --- |

**All tests**

| **A. Risk of Bias** | |
| --- | --- |
| Were the index test results interpreted without knowledge of the results of the reference standard? |  |
| If a threshold was used, was it pre-specified? |  |
| **Could the conduct or interpretation of the index test have introduced bias?** |  |

| **B. Concerns regarding applicability** | |
| --- | --- |
| **Are there concerns that the index test, its conduct, or interpretation differ from the review question?** |  |

**Reference Standard**

| **A. Risk of Bias** | | |
| --- | --- | --- |
| Target condition and reference standard(s) | Clinical diagnosis using Petersen's criteria and neurocognitive tests | |
| Is the reference standards likely to correctly classify the target condition? | |  |
| Were the reference standard results interpreted without knowledge of the results of the index tests? | |  |
| **Could the reference standard, its conduct, or its interpretation have introduced bias?** | |  |

| **B. Concerns regarding applicability** | |
| --- | --- |
| **Are there concerns that the target condition as defined by the reference standard does not match the question?** |  |

**Flow and Timing**

| **A. Risk of Bias** | | |
| --- | --- | --- |
| Flow and timing |  | |
| Was there an appropriate interval between index test and reference standard? | |  |
| Did all patients receive the same reference standard? | |  |
| Were all patients included in the analysis? | |  |
| **Could the patient flow have introduced bias?** | |  |

**Notes**

| **Notes** |  |
| --- | --- |

**Kluger 2009**

**Patient Selection**

| **A. Risk of Bias** | | |
| --- | --- | --- |
| Patient Sampling | academic referral practice, Control participants included spouses and caretakers of individuals who were seen at our Memory Disorders Clinic, as well as volunteers from the community | |
| Was a consecutive or random sample of patients enrolled? | |  |
| Was a case-control design avoided? | |  |
| Did the study avoid inappropriate exclusions? | |  |
| **Could the selection of patients have introduced bias?** | |  |

| **B. Concerns regarding applicability** | | |
| --- | --- | --- |
| Patient characteristics and setting | Control participants included spouses and caretakers of individuals attending the memory clinic | |
| **Are there concerns that the included patients and setting do not match the review question?** | |  |

**Index Test**

| Index tests | No name |
| --- | --- |

**All tests**

| **A. Risk of Bias** | |
| --- | --- |
| Were the index test results interpreted without knowledge of the results of the reference standard? |  |
| If a threshold was used, was it pre-specified? |  |
| **Could the conduct or interpretation of the index test have introduced bias?** |  |

| **B. Concerns regarding applicability** | |
| --- | --- |
| **Are there concerns that the index test, its conduct, or interpretation differ from the review question?** |  |

**Reference Standard**

| **A. Risk of Bias** | | |
| --- | --- | --- |
| Target condition and reference standard(s) | Clinical diagnosis using battery of neurocognitive tests | |
| Is the reference standards likely to correctly classify the target condition? | |  |
| Were the reference standard results interpreted without knowledge of the results of the index tests? | |  |
| **Could the reference standard, its conduct, or its interpretation have introduced bias?** | |  |

| **B. Concerns regarding applicability** | |
| --- | --- |
| **Are there concerns that the target condition as defined by the reference standard does not match the question?** |  |

**Flow and Timing**

| **A. Risk of Bias** | | |
| --- | --- | --- |
| Flow and timing |  | |
| Was there an appropriate interval between index test and reference standard? | |  |
| Did all patients receive the same reference standard? | |  |
| Were all patients included in the analysis? | |  |
| **Could the patient flow have introduced bias?** | |  |

**Notes**

| **Notes** |  |
| --- | --- |

**Lichtenberg 2006**

**Patient Selection**

| **A. Risk of Bias** | | |
| --- | --- | --- |
| Patient Sampling |  | |
| Was a consecutive or random sample of patients enrolled? | |  |
| Was a case-control design avoided? | |  |
| Did the study avoid inappropriate exclusions? | |  |
| **Could the selection of patients have introduced bias?** | |  |

| **B. Concerns regarding applicability** | | |
| --- | --- | --- |
| Patient characteristics and setting |  | |
| **Are there concerns that the included patients and setting do not match the review question?** | |  |

**Index Test**

| Index tests | CST |
| --- | --- |

**All tests**

| **A. Risk of Bias** | |
| --- | --- |
| Were the index test results interpreted without knowledge of the results of the reference standard? |  |
| If a threshold was used, was it pre-specified? |  |
| **Could the conduct or interpretation of the index test have introduced bias?** |  |

| **B. Concerns regarding applicability** | |
| --- | --- |
| **Are there concerns that the index test, its conduct, or interpretation differ from the review question?** |  |

**Reference Standard**

| **A. Risk of Bias** | | |
| --- | --- | --- |
| Target condition and reference standard(s) | Clinical diagnosis using Petersen's criteria; Clinical diagnosis of dementia using DSM-V | |
| Is the reference standards likely to correctly classify the target condition? | |  |
| Were the reference standard results interpreted without knowledge of the results of the index tests? | |  |
| **Could the reference standard, its conduct, or its interpretation have introduced bias?** | |  |

| **B. Concerns regarding applicability** | |
| --- | --- |
| **Are there concerns that the target condition as defined by the reference standard does not match the question?** |  |

**Flow and Timing**

| **A. Risk of Bias** | | |
| --- | --- | --- |
| Flow and timing |  | |
| Was there an appropriate interval between index test and reference standard? | |  |
| Did all patients receive the same reference standard? | |  |
| Were all patients included in the analysis? | |  |
| **Could the patient flow have introduced bias?** | |  |

**Notes**

| **Notes** |  |
| --- | --- |

**Maruff 2013**

**Patient Selection**

| **A. Risk of Bias** | | |
| --- | --- | --- |
| Patient Sampling | Consecutive | |
| Was a consecutive or random sample of patients enrolled? | |  |
| Was a case-control design avoided? | |  |
| Did the study avoid inappropriate exclusions? | |  |
| **Could the selection of patients have introduced bias?** | |  |

| **B. Concerns regarding applicability** | | |
| --- | --- | --- |
| Patient characteristics and setting | Primary care | |
| **Are there concerns that the included patients and setting do not match the review question?** | |  |

**Index Test**

| Index tests | CBB |
| --- | --- |

**All tests**

| **A. Risk of Bias** | |
| --- | --- |
| Were the index test results interpreted without knowledge of the results of the reference standard? |  |
| If a threshold was used, was it pre-specified? |  |
| **Could the conduct or interpretation of the index test have introduced bias?** |  |

| **B. Concerns regarding applicability** | |
| --- | --- |
| **Are there concerns that the index test, its conduct, or interpretation differ from the review question?** |  |

**Reference Standard**

| **A. Risk of Bias** | | |
| --- | --- | --- |
| Target condition and reference standard(s) | MCI and AD. Clinical, neuropsychological and structual neuroimaging | |
| Is the reference standards likely to correctly classify the target condition? | |  |
| Were the reference standard results interpreted without knowledge of the results of the index tests? | |  |
| **Could the reference standard, its conduct, or its interpretation have introduced bias?** | |  |

| **B. Concerns regarding applicability** | |
| --- | --- |
| **Are there concerns that the target condition as defined by the reference standard does not match the question?** |  |

**Flow and Timing**

| **A. Risk of Bias** | | |
| --- | --- | --- |
| Flow and timing | NR | |
| Was there an appropriate interval between index test and reference standard? | |  |
| Did all patients receive the same reference standard? | |  |
| Were all patients included in the analysis? | |  |
| **Could the patient flow have introduced bias?** | |  |

**Notes**

| **Notes** |  |
| --- | --- |

**Mundt 2001**

**Patient Selection**

| **A. Risk of Bias** | | |
| --- | --- | --- |
| Patient Sampling |  | |
| Was a consecutive or random sample of patients enrolled? | |  |
| Was a case-control design avoided? | |  |
| Did the study avoid inappropriate exclusions? | |  |
| **Could the selection of patients have introduced bias?** | |  |

| **B. Concerns regarding applicability** | | |
| --- | --- | --- |
| Patient characteristics and setting |  | |
| **Are there concerns that the included patients and setting do not match the review question?** | |  |

**Index Test**

| Index tests | Computer automated telephone screening |
| --- | --- |

**All tests**

| **A. Risk of Bias** | |
| --- | --- |
| Were the index test results interpreted without knowledge of the results of the reference standard? |  |
| If a threshold was used, was it pre-specified? |  |
| **Could the conduct or interpretation of the index test have introduced bias?** |  |

| **B. Concerns regarding applicability** | |
| --- | --- |
| **Are there concerns that the index test, its conduct, or interpretation differ from the review question?** |  |

**Reference Standard**

| **A. Risk of Bias** | | |
| --- | --- | --- |
| Target condition and reference standard(s) | Clinical diagnosis using CDR score | |
| Is the reference standards likely to correctly classify the target condition? | |  |
| Were the reference standard results interpreted without knowledge of the results of the index tests? | |  |
| **Could the reference standard, its conduct, or its interpretation have introduced bias?** | |  |

| **B. Concerns regarding applicability** | |
| --- | --- |
| **Are there concerns that the target condition as defined by the reference standard does not match the question?** |  |

**Flow and Timing**

| **A. Risk of Bias** | | |
| --- | --- | --- |
| Flow and timing |  | |
| Was there an appropriate interval between index test and reference standard? | |  |
| Did all patients receive the same reference standard? | |  |
| Were all patients included in the analysis? | |  |
| **Could the patient flow have introduced bias?** | |  |

**Notes**

| **Notes** |  |
| --- | --- |

**O'Connell 2004**

**Patient Selection**

| **A. Risk of Bias** | | |
| --- | --- | --- |
| Patient Sampling |  | |
| Was a consecutive or random sample of patients enrolled? | |  |
| Was a case-control design avoided? | |  |
| Did the study avoid inappropriate exclusions? | |  |
| **Could the selection of patients have introduced bias?** | |  |

| **B. Concerns regarding applicability** | | |
| --- | --- | --- |
| Patient characteristics and setting |  | |
| **Are there concerns that the included patients and setting do not match the review question?** | |  |

**Index Test**

| Index tests | CANTAB-PAL |
| --- | --- |

**All tests**

| **A. Risk of Bias** | |
| --- | --- |
| Were the index test results interpreted without knowledge of the results of the reference standard? |  |
| If a threshold was used, was it pre-specified? |  |
| **Could the conduct or interpretation of the index test have introduced bias?** |  |

| **B. Concerns regarding applicability** | |
| --- | --- |
| **Are there concerns that the index test, its conduct, or interpretation differ from the review question?** |  |

**Reference Standard**

| **A. Risk of Bias** | | |
| --- | --- | --- |
| Target condition and reference standard(s) | Clinical diagnosis using NINCDS-ADRDA criteria | |
| Is the reference standards likely to correctly classify the target condition? | |  |
| Were the reference standard results interpreted without knowledge of the results of the index tests? | |  |
| **Could the reference standard, its conduct, or its interpretation have introduced bias?** | |  |

| **B. Concerns regarding applicability** | |
| --- | --- |
| **Are there concerns that the target condition as defined by the reference standard does not match the question?** |  |

**Flow and Timing**

| **A. Risk of Bias** | | |
| --- | --- | --- |
| Flow and timing | Retrospective design | |
| Was there an appropriate interval between index test and reference standard? | |  |
| Did all patients receive the same reference standard? | |  |
| Were all patients included in the analysis? | |  |
| **Could the patient flow have introduced bias?** | |  |

**Notes**

| **Notes** |  |
| --- | --- |

**Rosenthal 2013**

**Patient Selection**

| **A. Risk of Bias** | | |
| --- | --- | --- |
| Patient Sampling |  | |
| Was a consecutive or random sample of patients enrolled? | |  |
| Was a case-control design avoided? | |  |
| Did the study avoid inappropriate exclusions? | |  |
| **Could the selection of patients have introduced bias?** | |  |

| **B. Concerns regarding applicability** | | |
| --- | --- | --- |
| Patient characteristics and setting |  | |
| **Are there concerns that the included patients and setting do not match the review question?** | |  |

**Index Test**

| Index tests | CAMCI Modified |
| --- | --- |

**All tests**

| **A. Risk of Bias** | |
| --- | --- |
| Were the index test results interpreted without knowledge of the results of the reference standard? |  |
| If a threshold was used, was it pre-specified? |  |
| **Could the conduct or interpretation of the index test have introduced bias?** |  |

| **B. Concerns regarding applicability** | |
| --- | --- |
| **Are there concerns that the index test, its conduct, or interpretation differ from the review question?** |  |

**Reference Standard**

| **A. Risk of Bias** | | |
| --- | --- | --- |
| Target condition and reference standard(s) | Clinical diagnosis using neurocognitive tests and Albert criteria and Peterson criteria for aMCI | |
| Is the reference standards likely to correctly classify the target condition? | |  |
| Were the reference standard results interpreted without knowledge of the results of the index tests? | |  |
| **Could the reference standard, its conduct, or its interpretation have introduced bias?** | |  |

| **B. Concerns regarding applicability** | |
| --- | --- |
| **Are there concerns that the target condition as defined by the reference standard does not match the question?** |  |

**Flow and Timing**

| **A. Risk of Bias** | | |
| --- | --- | --- |
| Flow and timing |  | |
| Was there an appropriate interval between index test and reference standard? | |  |
| Did all patients receive the same reference standard? | |  |
| Were all patients included in the analysis? | |  |
| **Could the patient flow have introduced bias?** | |  |

| **Notes** | The outcomes are not all reported |
| --- | --- |

**Saxton 2009**

**Patient Selection**

| **A. Risk of Bias** | | |
| --- | --- | --- |
| Patient Sampling | Patients were recruited through PCPs and community | |
| Was a consecutive or random sample of patients enrolled? | |  |
| Was a case-control design avoided? | |  |
| Did the study avoid inappropriate exclusions? | |  |
| **Could the selection of patients have introduced bias?** | |  |

| **B. Concerns regarding applicability** | | |
| --- | --- | --- |
| Patient characteristics and setting | Patients were recruited through PCPs and community | |
| **Are there concerns that the included patients and setting do not match the review question?** | |  |

**Index Test**

| Index tests | CAMCI |
| --- | --- |

**All tests**

| **A. Risk of Bias** | |
| --- | --- |
| Were the index test results interpreted without knowledge of the results of the reference standard? |  |
| If a threshold was used, was it pre-specified? |  |
| **Could the conduct or interpretation of the index test have introduced bias?** |  |

| **B. Concerns regarding applicability** | |
| --- | --- |
| **Are there concerns that the index test, its conduct, or interpretation differ from the review question?** |  |

**Reference Standard**

| **A. Risk of Bias** | | |
| --- | --- | --- |
| Target condition and reference standard(s) | Clinical diagnosis using battery of neurocognitive tests | |
| Is the reference standards likely to correctly classify the target condition? | |  |
| Were the reference standard results interpreted without knowledge of the results of the index tests? | |  |
| **Could the reference standard, its conduct, or its interpretation have introduced bias?** | |  |

| **B. Concerns regarding applicability** | |
| --- | --- |
| **Are there concerns that the target condition as defined by the reference standard does not match the question?** |  |

**Flow and Timing**

| **A. Risk of Bias** | | |
| --- | --- | --- |
| Flow and timing | 10% attrition reasons stated no details of the timing | |
| Was there an appropriate interval between index test and reference standard? | |  |
| Did all patients receive the same reference standard? | |  |
| Were all patients included in the analysis? | |  |
| **Could the patient flow have introduced bias?** | |  |

|  |  |
| --- | --- |

**Tierney 2014**

**Patient Selection**

| **A. Risk of Bias** | | |
| --- | --- | --- |
| Patient Sampling |  | |
| Was a consecutive or random sample of patients enrolled? | |  |
| Was a case-control design avoided? | |  |
| Did the study avoid inappropriate exclusions? | |  |
| **Could the selection of patients have introduced bias?** | |  |

| **B. Concerns regarding applicability** | | |
| --- | --- | --- |
| Patient characteristics and setting |  | |
| **Are there concerns that the included patients and setting do not match the review question?** | |  |

**Index Test**

| Index tests | CAMCI |
| --- | --- |

**All tests**

| **A. Risk of Bias** | |
| --- | --- |
| Were the index test results interpreted without knowledge of the results of the reference standard? |  |
| If a threshold was used, was it pre-specified? |  |
| **Could the conduct or interpretation of the index test have introduced bias?** |  |

| **B. Concerns regarding applicability** | |
| --- | --- |
| **Are there concerns that the index test, its conduct, or interpretation differ from the review question?** |  |

**Reference Standard**

| **A. Risk of Bias** | | |
| --- | --- | --- |
| Target condition and reference standard(s) | Clinical diagnosis using battery of neurocognitive tests | |
| Is the reference standards likely to correctly classify the target condition? | |  |
| Were the reference standard results interpreted without knowledge of the results of the index tests? | |  |
| **Could the reference standard, its conduct, or its interpretation have introduced bias?** | |  |

| **B. Concerns regarding applicability** | |
| --- | --- |
| **Are there concerns that the target condition as defined by the reference standard does not match the question?** |  |

**Flow and Timing**

| **A. Risk of Bias** | | |
| --- | --- | --- |
| Flow and timing |  | |
| Was there an appropriate interval between index test and reference standard? | |  |
| Did all patients receive the same reference standard? | |  |
| Were all patients included in the analysis? | |  |
| **Could the patient flow have introduced bias?** | |  |

|  |  |
| --- | --- |

**Vacante 2013**

**Patient Selection**

| **A. Risk of Bias** | | |
| --- | --- | --- |
| Patient Sampling | Consecutive | |
| Was a consecutive or random sample of patients enrolled? | |  |
| Was a case-control design avoided? | |  |
| Did the study avoid inappropriate exclusions? | |  |
| **Could the selection of patients have introduced bias?** | |  |

| **B. Concerns regarding applicability** | | |
| --- | --- | --- |
| Patient characteristics and setting |  | |
| **Are there concerns that the included patients and setting do not match the review question?** | |  |

**Index Test**

| Index tests | TPT |
| --- | --- |

**All tests**

| **A. Risk of Bias** | |
| --- | --- |
| Were the index test results interpreted without knowledge of the results of the reference standard? |  |
| If a threshold was used, was it pre-specified? |  |
| **Could the conduct or interpretation of the index test have introduced bias?** |  |

| **B. Concerns regarding applicability** | |
| --- | --- |
| **Are there concerns that the index test, its conduct, or interpretation differ from the review question?** |  |

**Reference Standard**

| **A. Risk of Bias** | | |
| --- | --- | --- |
| Target condition and reference standard(s) | Clinical diagnosis using Petersen's criteria | |
| Is the reference standards likely to correctly classify the target condition? | |  |
| Were the reference standard results interpreted without knowledge of the results of the index tests? | |  |
| **Could the reference standard, its conduct, or its interpretation have introduced bias?** | |  |

| **B. Concerns regarding applicability** | |
| --- | --- |
| **Are there concerns that the target condition as defined by the reference standard does not match the question?** |  |

**Flow and Timing**

| **A. Risk of Bias** | | |
| --- | --- | --- |
| Flow and timing |  | |
| Was there an appropriate interval between index test and reference standard? | |  |
| Did all patients receive the same reference standard? | |  |
| Were all patients included in the analysis? | |  |
| **Could the patient flow have introduced bias?** | |  |
